# Rhein alleviates renal interstitial fibrosis by inhibiting tubular cell apoptosis in rats

**DOI:** 10.1186/s40659-019-0257-0

**Published:** 2019-09-06

**Authors:** Yakun Chen, Lin Mu, Lingling Xing, Shaomei Li, Shuxia Fu

**Affiliations:** Renal Division, Department of Medicine, The Second Hospital of Hebei Medical University, Shijiazhuang, 050000 Hebei China

**Keywords:** Rhein, Renal interstitial fibrosis, Apoptosis, Unilateral ureteral obstruction, Collagen

## Abstract

**Background:**

Ureteral obstruction causes injury of the renal tissues and can irreversibly progress to renal fibrosis, with atrophy and apoptosis of tubular cells. The goal of the current study was to examine the effects of rhein on the apoptosis o renal tubular cells as well as renal fibrosis using a rodent model of unilateral ureteral obstruction (UUO).

**Methods:**

UUO was induced through ureteral ligation, then animals received treatments with rhein or vehicle. The control rats only received sham operation. The renal tissue was harvested 1 week after surgery for assessment of kidney fibrosis.

**Results:**

The expressions of collagen I and α-smooth muscle actin (α-SMA), as well as the severity of renal tubular apoptosis and fibrosis were time-dependently increased following UUO. Treatments with rhein partially inhibited such responses. Renal interstitial fibrosis was associated with STAT3 (signal transducer and activator of transcription 3) phosphorylation as well as altered expressions of Bax and Bcl2, both apoptosis-related proteins. Treatment with rhein also partly blocked these responses.

**Conclusion:**

These findings demonstrated that rhein mitigated apoptosis of renal tubular cell as well as renal fibrosis in a UUO rodent model. This curative effect is likely mediated via suppression of STAT3 phosphorylation.

## Background

Chronic kidney disease (CKD) is caused by different types of kidney injuries, affecting around 10% of the global population [1]. CKD is a progressive condition with characteristics such as rising severity of renal interstitial fibrosis [1, 2]. In every type of end-stage renal diseases, renal tubulointerstitial fibrosis has been regarded as a common final stage with abnormal renal fibroblasts growth and activation [1, 2]. The active fibroblast can be regarded a myofibroblast based on phenotypic changes, such as elevated levels of extracellular matrix (ECM) components, i.e. fibronectin, and α-smooth muscle actin (α-SMA) [1–3]. Insights into the molecular mechanisms underlying the renal fibroblasts activation may inspire novel strategies in treating progressive renal conditions.

Characteristics of unilateral ureteral obstruction (UUO) include apoptosis of tubular cell, renal interstitial fibrosis, and progressive renal atrophy [4]. A number of signal transduction molecules and pathways are involved in renal interstitial fibroblasts activation [2, 5]. STAT3 (Signal transducer and activator of transcription 3) was reported to be enriched in interstitial fibroblasts after UUO-induced injuries [6]. In addition, STAT3 phosphorylation contributes to apoptosis of renal tubular cell induced by injury induced by ischemia–reperfusion [7, 8]. By far, seven members have been identified in the STAT family, namely STAT1-4, 5a, 5b and 6, mediating diverse functions such as cell proliferation and survival [9, 10]. It is demonstrated in a prior investigation that activation of STAT3 is enhanced in interstitial fibroblasts after UUO through phosphorylation at tyrosine-705 [6]. The actions of STAT3 in renal interstitial fibroblasts activation and tubular cells apoptosis have not been evaluated in vivo yet.

UUO has detrimental impacts on the kidney, contributing to the majority of kidney insufficiency observed in both young and old [11]. Despite surgical management without delay, obstruction of urinary tract can still cause renal unit damage that is irreversible [12]. Hence, pharmacological approach to manage obstructive nephropathy is of great importance as well. The renin-angiotensin system (RAS), active during renal development in the developing kidney, is activated by UUO as well [3]. There remains controversy as to whether blockage of RAS alleviates apoptosis of renal tubular epithelial cell as well as renal interstitial fibrosis [12–15].

Rhubarb, a popular traditional Chinese herbal medicine, is widely utilized in traditional Chinese medicine to treat renal diseases. The clinical efficacy of rhubarb was previously established, however, the mechanism underlying its effect against renal diseases was not yet clear. Rhein is a most bioactive component in rhubarb. Previous work has described rhein to potently inhibit hepatic fibrosis induced by carbon tetrachloride, one compound that could reduce a-SMA expression and collagen synthesis [16]. Rhein has been implicated in various animal models of renal diseases. For instance, in mice with renal fibrosis phenotype, rhein was able to ameliorate symptoms by reversing DNA hypermethylation-associated Klotho suppression [17]. Rhein also inhibited autophagy in rat renal tubular cells by regulating the AMPK/mTOR pathway [18].

The current study assessed the curative effect of rhein on the apoptosis of tubular cell and development of renal fibrosis using a rat kidney fibrosis model. Further, the impact of rhein on STAT3 phosphorylation, a critical component in the apoptosis of tubular cell as well as progression of renal fibrosis, was examined.

## Materials and methods

### Establish of UUO rat model and rhein treatment

Sprague–Dawley rats (all males) with body weight around 170–210 g were kept at the Animal Facility of the Second Hospital of Hebei Medical University under a 11 h:13 h light/dark cycle (lights on at 07:00 and off at 18:00) with a constant temperature (18 ± 1 ˚C) and ad libitum access to food and water. The animal protocol was approved by the Animal Care and Use Committee of the Second Hospital of Hebei Medical University.

Rats were divided randomly into three groups with 10 rats in each group: sham, UUO with vehicle control (UUO + veh) and UUO with rhein therapy group (UUO + rhein). Rhein (catalog no. 30873, Sigma, St. Louis, MO, USA) was dissolved in 20% ethanol. Rats were given a daily dose of rhein at 150 mg/kg or vehicle at equal volume after UUO operation until the end of experiments. For UUO operation, rats were deeply anesthetized using pentobarbital (50 mg per kg body weight) injected intraperitoneally (i.p). A midline incision was made to expose the abdominal cavity, and the left ureter was separated to be ligated. The animals in the sham group were subjected to identical surgical procedures except for the ureter ligation. Rats were sacrificed 1 week following the ureter ligation. After all experiments, animals were deeply anaesthetized with i.p injection of xylazine (10 mg/kg) and ketamine (75 mg/kg). Kidney samples were then collected for histological analysis or Western blot assay.

## Renal morphology

After fixation using 4% buffered formalin and dehydration through a graded ethanol series, the renal tissue was embedded in paraffin and transverse sections were made on a microtome at 4 μm thickness, which was then stained using Masson's trichrome. Examination of renal morphology was performed under microscopy (Olympus, Japan). Counting of Masson's trichrome-positive areas was used to determine the levels of the renal fibrosis. Blue linear or granular deposits were counted as collagen positive. Staining quantification were performed using MetaMorph/C-5050/BX41 image analysis program (Olympus, Japan), and calculated as the proportion of total stained area.

## Western blot

After three washes with phosphate-buffered saline (PBS), tissue samples were prepared into a suspension in radioimmunoprecipitation assay buffer (Biyuntian, Shanghai, China) using a tissue homogenizer, followed by centrifugation at 10,000×*g* for 20 min at 4 ˚C, and then the resulted supernatants were kept at -80 ˚C for further analysis. The concentration of protein was measured using the bicinchoninic acid protein assay (Biyuntian, Shanghai, China). All samples were adjusted to 40 μg total protein, then denatured in loading buffer [10% glycerol, 62 mM Tris, 0.003% bromophenol blue, 2% sodium dodecyl sulfate (SDS), pH 7.4], separated on an SDS–polyacrylamide gel (8%) at 100 V for 2 h, and subsequently transferred to polyvinylidene difluoride-plus membranes (60 min at 80 V; MSI, Westborough, MA, USA) using a semi-dry protein transfer system (UVP Inc., Upland, CA, USA). Bovine serum albumin (5%) or 5% non-fat dry milk in Tris-buffered saline (TBS; pH 7.4) with 0.2% Tween-20 (TBST) was used to block the membranes at room temperature for 1 h, which was subjected to three washes in TBST (10 min each), and subsequent overnight incubation at 4 ˚C with one of the following primary antibodies: anti-STAT3, anti-phospho-STAT3 (p-STAT3) (both 1:1000 dilution; Cell Signalling Technology, Danvers, MA, USA), anti-Bcl2, anti-Bax (both 1:500 dilution; Santa Cruz Biotechnology, Santa Cruz, CA, USA), anti-type I collagen, and anti-α-SMA (both 1:1,000 dilution; Sigma, St. Louis, MO, USA). Next, the membranes were again rinsed in TBST for three times, followed by incubation with appropriate HRP-conjugated secondary antibody at room temperature for 2 h, followed by another three washes (10 min each) in TBST. Final visualization of bands was performed with the aid of an enhanced chemiluminescence kit (Amersham, NJ, USA). GAPDH (1:1000 dilution; Santa Cruz, CA, USA) was included as the loading control. The concentrations of protein were determined with an image analysis program (Bio-Rad, MD, USA).

### Terminal deoxynucleotidyl transferase-mediated dUTP nick end labeling (TUNEL) assay

The quantity of cell nuclei with fragmented DNA, for instance apoptotic cells, was determined using the TUNEL assay kit (Roche Diagnostics, Mannheim, Germany). After deparaffinization and hydration, tissue sections were washed in PBS for three times (10 min each). Treatment with 0.3% hydrogen peroxide in methanol for 30 min was used to quench endogenous peroxidase, and 10 μg/ml proteinase-K was used to treat the sections for 15 min, which were then rinsed with PBS, followed by 1 h incubation with digoxigenin-dNTP and deoxynucleotidyl transferase. After termination of the reaction, the sections were rinsed with PBS again, then added with anti-digoxigenin antibody at ambient temperature and allowed to react for 30 min. After another 3 rinses with PBS, the sections were developed with 3,3′-diaminobenzidine, followed by counterstaining with hematoxylinn (10%). Apoptosis-positive nuclei were manually counted at ×400 magnification from ten random fields. The numbers of apoptotic cells in the interstitium as well as tubules were pooled. In every group, the apoptosis-positive nuclei distributed approximately evenly in the interstitial and tubular cells.

### Statistical analysis

All results were presented as the means ± standard deviation (SD). Statistics was performed using the SPSS version 10.5 (Chicago, IL, USA). Sufficiency of animal group size was verified by Cohen’s d method [19]. The means of data from all groups were divided by their standard deviation to calculate the standardized effect size, the largest of which was then referenced to Cohen’s d power table to determine minimum group size [6], compared to which our group size of 10 is sufficient. Comparisons of mean values were conducted using a one-way analysis of variance with Dunnett’s multiple comparison test. P values less than 0.05 were considered statistically significant.

## Results

### UUO increases the expression of α-SMA and type I collagen and TUNEL staining

**Fig. 1:**
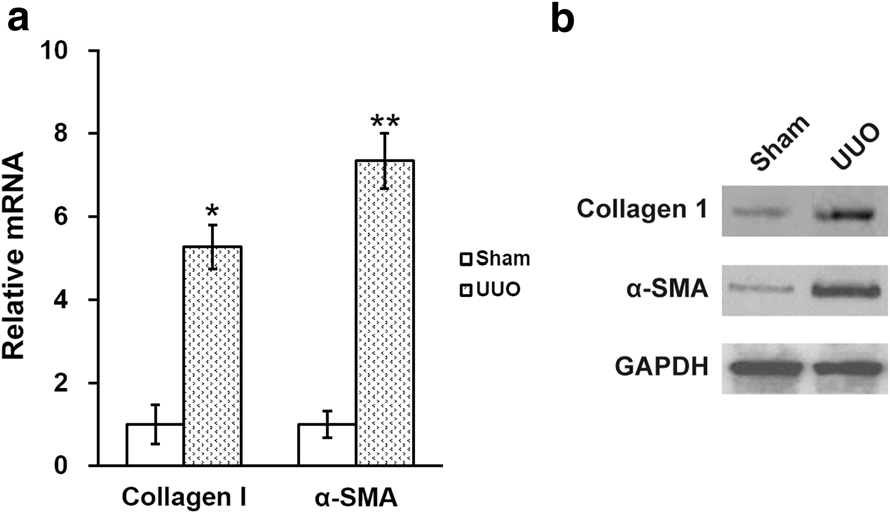
Effect of UUO on the expression levels of renal type I collagen and α-SMA in the rat kidneys. mRNA (**a**) and protein (**b**) expression levels of renal type I collagen and α-SMA in the kidneys of sham and UUO model rats. Data were shown as mean ± SD. *p < 0.05, **p < 0.01, compared to sham

The expression of α-SMA and type I collagen in the kidneys from both sham control and UUO rats were evaluated. α-SMA, a tubulointerstitial myofibroblasts bio-marker, contributes to a prominent portion of the UUO-induced interstitial collagen deposition. Western blot analysis as well as RT-PCR indicated that both protein and transcript levels of α-SMA in the UUO rats were markedly higher than that in the sham group (Fig. 1a, b). The mRNA and protein levels of collagen I also displayed similar trends (Fig. 1a, b).

**Fig. 2:**
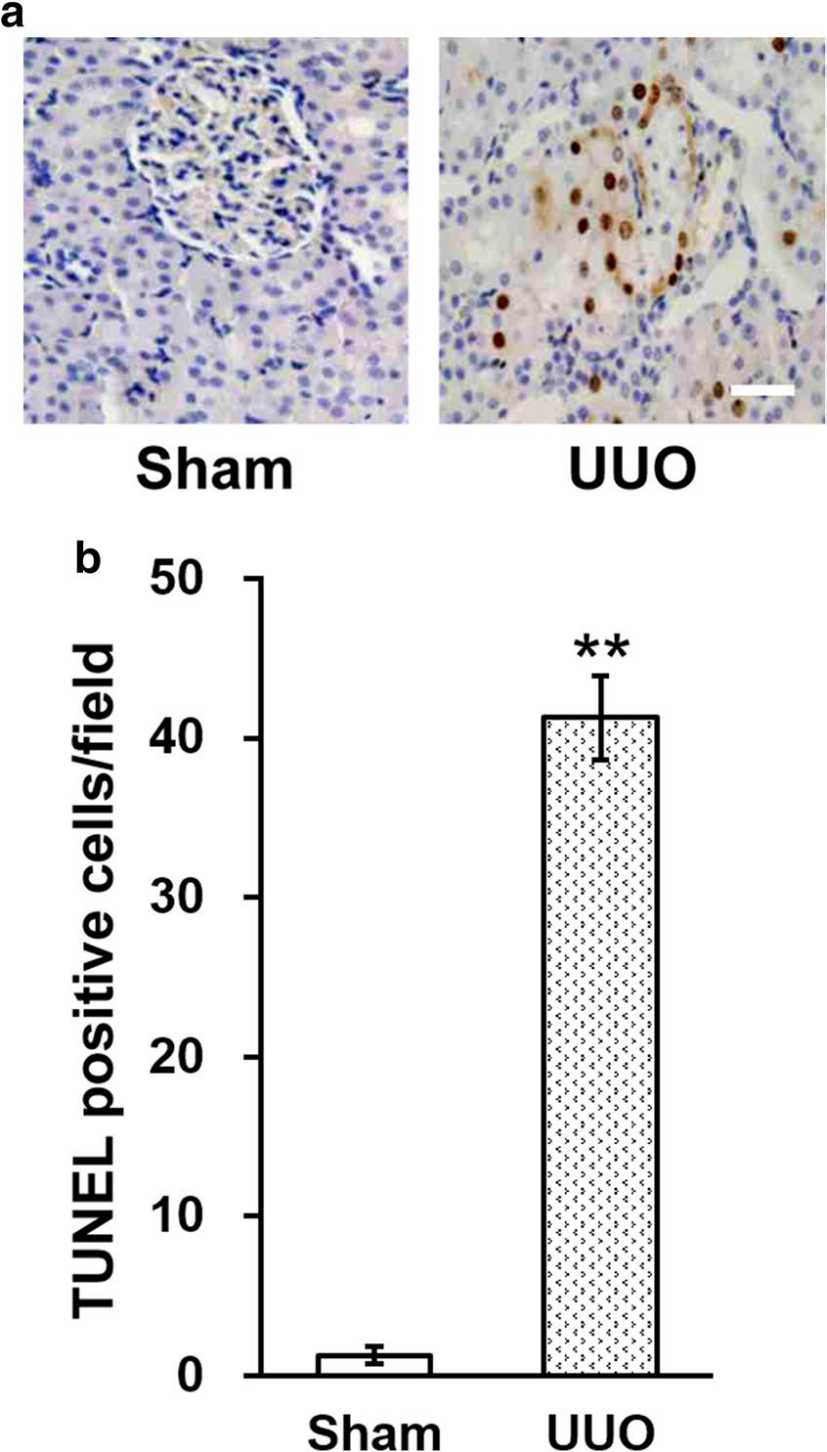
Effect of UUO on the levels of renal tubular apoptosis (TUNEL assay). **a** Representative images of TUNEL staining in the kidneys of sham and UUO model rats, scale bar 50 μm. **b** Quantitation of the TUNEL staining results in **a**. Data were shown as mean ± SD. **p < 0.01, compared to sham

The TUNEL assay demonstrated that apoptotic tubular cells were scarse in renal tissues of the sham-operated rats (Fig. 2a). However, the extent of tubular apoptosis are elevated following UUO (Fig. 2a), and the quantity of TUNEL-positive cells was indeed found to be significantly increased in UUO model rats compared to that of sham rats (Fig. 2b).

### Rhein attenuates STAT3 phosphorylation in the obstructed kidney

**Fig. 3:**
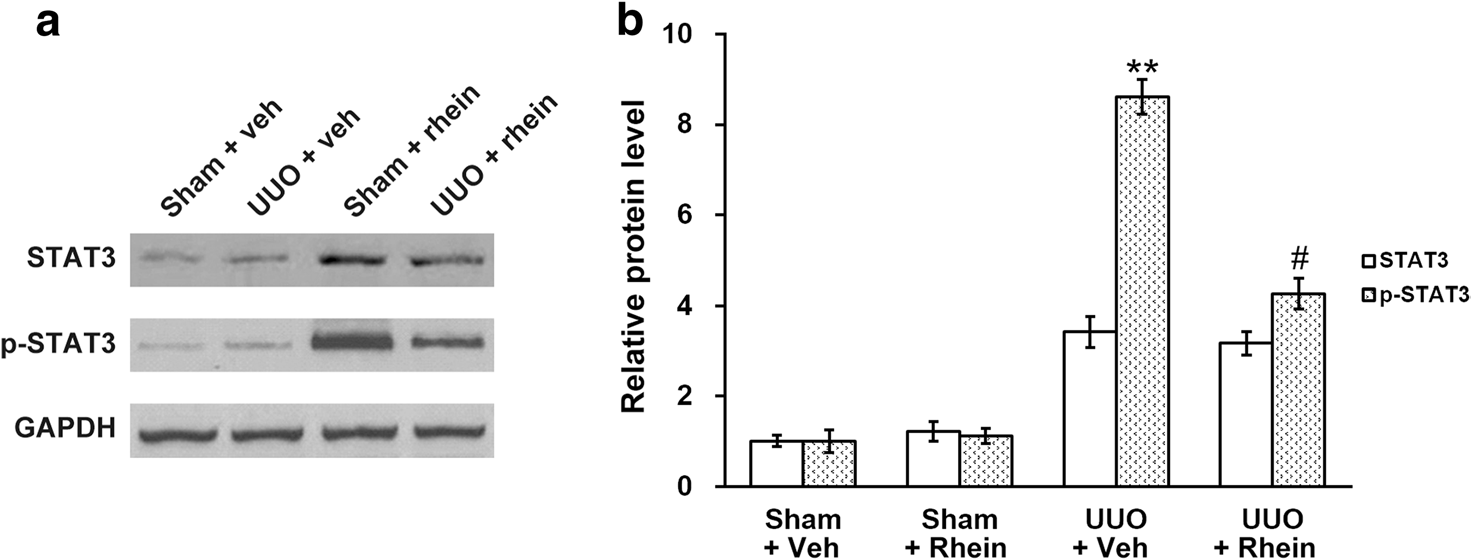
Effect of rhein on the phosphorylation levels of STAT3 in the kidney of UUO model rats. **a** Western blot analysis of the kidney tissue lysates of sham and UUO rats with vehicle (veh) or rhein treatment, using antibodies against STAT3, p-STAT3 and GAPDH (as loading control). **b** Quantification of the Western blot results in **a** by densitometry, expressed as fold-increase over the controls. Data were shown as mean ± SD. **p < 0.01, compared to UUO + veh/STAT3. ^#^p < 0.05, compared to UUO + veh/p-STAT3

Prior investigations have shown in the interstitial fibroblasts of fibrotic kidneys there is upregulation of phosphorylated STAT3 [2, 6]. In line with these previous findings, it was found in the current study that p-STAT3 levels also increased in obstructed kidneys (Fig. 3a). The effects of rhein on the expression and phosphorylation of STAT3 in the obstructed kidneys was then assessed. The data demonstrated that rhein treatment markedly suppressed UUO-induced p-STAT3, but not yet to sham control levels (Fig. 3a, b). UUO injury also elicited upregulation of STAT3 expression, however, rhein did not affect overall STAT3 expression level (Fig. 3a, b).

### Rhein attenuates progression of renal fibrosis

**Fig. 4:**
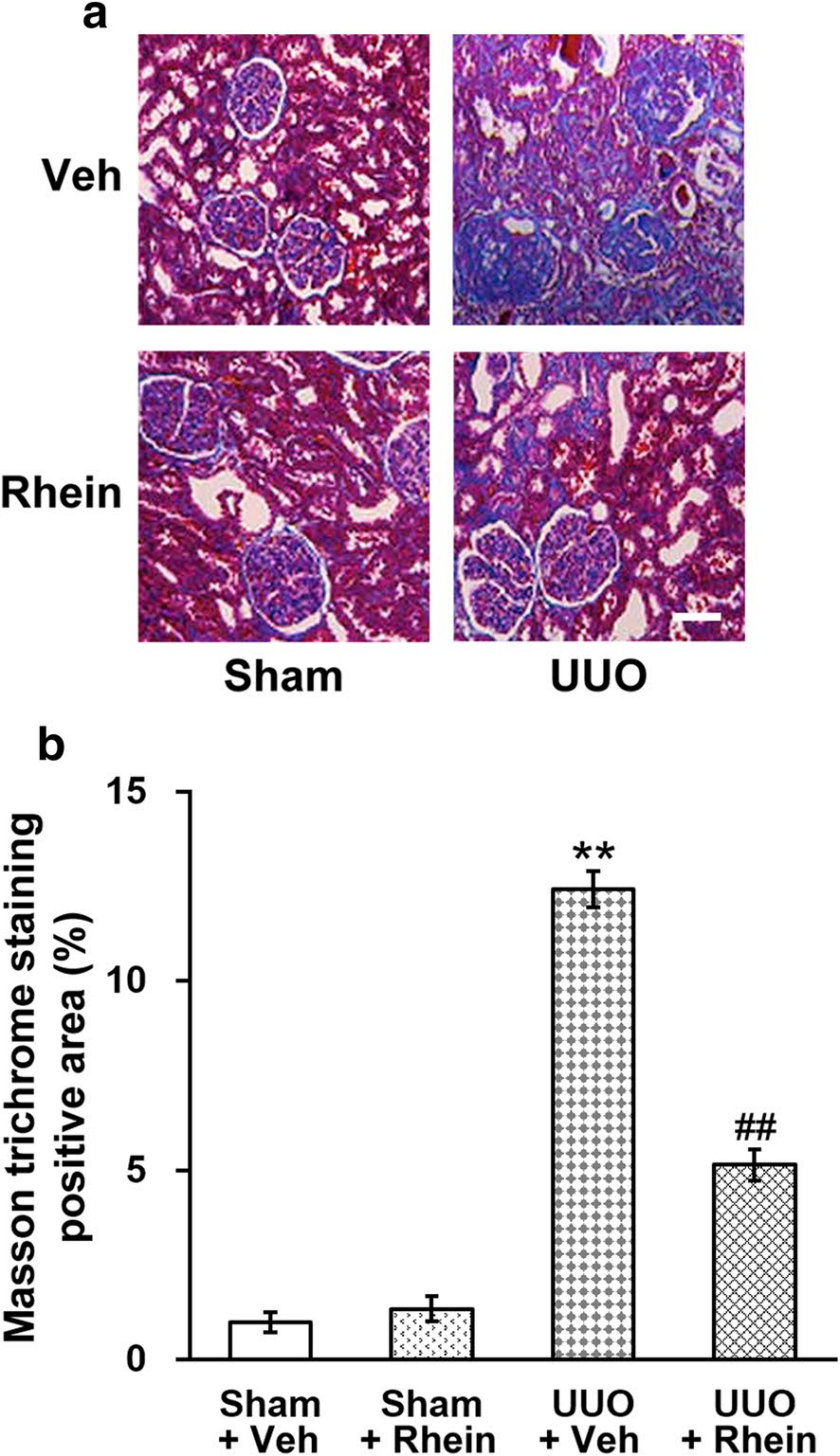
Effect of rhein on renal fibrosis in UUO model rats. **a** Representative images of Masson trichrome staining of sham and UUO rats with vehicle (veh) or rhein treatment, scale bar 50 μm. **b** Quantification of Masson trichrome staining results. Data were shown as mean ± SD. **p < 0.01, compared to sham + veh and sham + rhein. ^##^p < 0.01, compared to UUO + veh

Next, we examined the effects of rhein on interstitial fibrosis using Masson's trichrome staining. The kidneys from the sham group had cuboid-shaped epithelial cells, no apparent interstitium, and back-to-back tubules. However, in obstructed kidneys, significant higher levels of blue staining were observed, suggesting interstitial fibrosis. Further, the adjacent tubules were separated. Rhein administration dramatically prevented the fibrosis, but not to control levels (Fig. 4a). Quantified results showed that the interstitial volume was low in the sham kidneys, and was significantly elevated in UUO kidneys, and was then partially restored in the kidneys with UUO injuries and rhein treatment (Fig. 4b).

**Fig. 5:**
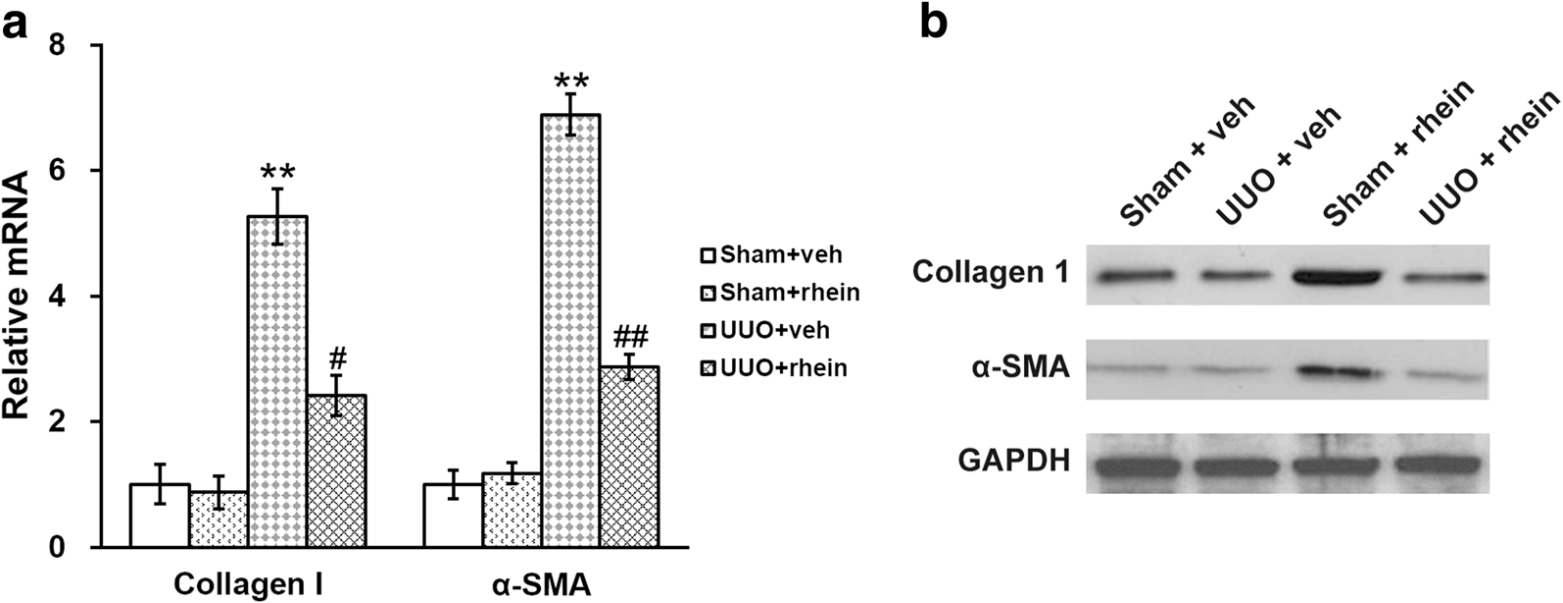
Effect of rhein on the expression levels of renal type I collagen and α-SMA in the UUO model rats. mRNA (**a**) and protein (**b**) expression levels of renal type I collagen and α-SMA in the kidneys of sham and UUO rats with vehicle (veh) or rhein treatment. Data were shown as mean ± SD. **p < 0.01, compared to sham + veh and sham + rhein. ^#^p < 0.05, ^##^p < 0.01, compared to UUO + veh

The expression of α-SMA and type I collagen in the kidneys of vehicle- and rhein-treated rats were examined as well (Fig. 5). The transcript levels of α-SMA as well as type I collagen were markedly higher in the kidneys with UUO injuries, and such effect was partially blocked by treatment with rhein (Fig. 5a). Protein levels of α-SMA and type I collagen followed the same trend as their mRNA in all four experimental groups of rats (Fig. 5b).

### Rhein attenuates tubular cell apoptosis and expression of apoptotic proteins following UUO

**Fig. 6:**
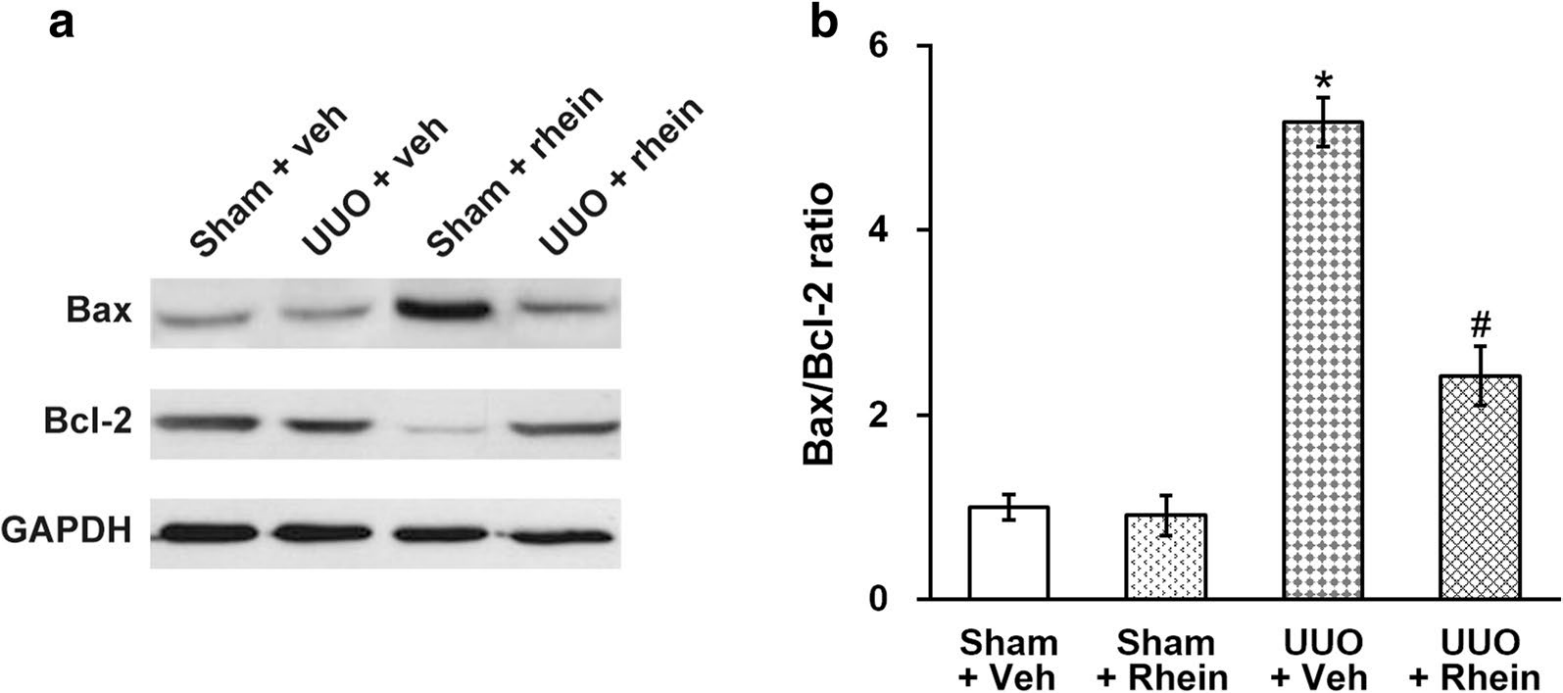
Effect of rhein on the levels of renal tubular cell apoptosis following UUO injury. **a** Western blot analysis of the kidney tissue lysates of sham and UUO rats with vehicle (veh) or rhein treatment, using antibodies against Bax, Bcl-2 and GAPDH (as loading control). **b** Quantification of the Western blot results in (A) by densitometry, expressed as Bax/Bcl-2 ratio. *p < 0.05, compared to sham + veh and sham + rhein. ^#^p < 0.05, compared to UUO + veh

In the renal tissues following the sham surgical operation no evident signs of apoptotic cell death were found (Fig. 2), whereas following UUO, a great amount of cells positive for TUNEL staining in the renal tubules were observed. We therefore employed Western blot to assess the expression of Bax (an apoptosis promoter) as well as Bcl2 (an apoptosis inhibitor), two important proteins involved in apoptosis (Fig. 6a). In the kidneys following UUO, the expression of Bcl2 was lower, while the expression of Bax was elevated compared to the control. We also calculated Bax/Bcl2 ratio, one of the molecular parameters commonly utilized to evaluate tissue damage in kidney or other pathologies (Fig. 6b), and found that the Bax/Bcl2 ratio was greatly escalated by UUO, and was then significantly reduced by rhein treatment, although not yet fully restored to the sham levels.

## Discussion

The characteristics of UUO include renal interstitial fibrosis, tubular cell apoptosis, and progressive renal atrophy [4]. Activity of RAS are elevated during the primary periods of renal injuries, and potentiated by UUO [13]. The findings of this current report revealed that rhein attenuates renal fibrosis following UUO, as evidenced by decreased expression of type I collagen and deposition of ECM. The cellular events through which rhein prevented renal fibrosis, however, are not entirely clear. An earlier study showed that α-SMA-positive myofibroblasts are predominantly responsible for over production of ECM in the kidney with fibrosis [2]; hence, the current investigation assessed the impact of rhein on α-SMA activation in vivo. Our findings indicated that rhein administration partially prevented the interstitial fibrosis and suppressed type I collagen and α-SMA expression after UUO.

Renal fibrosis has been shown to be a complex process involving an orchestral interaction of various molecular and cellular factors [2, 20]. The Janus kinase (JAK)/STAT signaling carry an essential function in the pathogenesis of various renal conditions. It is generally believed that JAK is activated by growth hormones or cytokines binding to their receptors, and in turn induces phosphorylation of the receptor at the intracellular domain and allows the phosphorylation and recruitment of STAT [21]. Phosphorylated STATs translocate to the nucleus, in the form of homo- or hetero-dimers, to activate the transcription of target genes [21]. Prior investigations have demonstrated that activities of the STAT3 signaling contribute to the progression of renal fibrosis in the UUO model [6, 22]. Consistently, it was demonstrated in the current study that STAT3 phosphorylation is induced by UUO in the kidney with fibrosis. The persistent and prominent increase of p-STAT3 implicated a important role of this protein in progression of renal fibrosis after UUO and interstitial fibroblasts activation. The results also undoubtedly showed that treatment with rhein partly inhibited the UUO induced STAT3 phosphorylation as well as renal fibrosis.

Obstruction of upper urinary tract results in injuries to the renal tissues as well as apoptosis of tubular cells, and subsequent rise of chemokines and cytokines released from the apoptotic tubular cells which leads to interstitial fibrosis and inflammatory responses [23]. Hence, suppression of apoptotic tubular cell may protect against the inflammatory signals generated from apoptotic cells, thereby attenuating the accumulated proapoptotic and profibrotic cytokines in the kidney and the following inflammation [24]. The current data also showed that the UUO-induced renal injury generated an increase of the Bax/Bcl2 ratio (a pro-apoptotic signal) following renal obstruction, and that prolonged UUO injury resulted in elevated levels of tubular cell atrophy and apoptosis. Furthermore, rhein partly attenuated the apoptosis associated with unilateral obstructive nephropathy, which was further supported by the observation that rhein had inhibitory effects on the activation of STAT3 signaling. Data of our current study is consistent with those of Zhao et al. [25], in which rhein shows a protective effect against cerebral ischemic- or reperfusion-induced oxidative stress and apoptosis in rats, suggesting the anti-apoptosis activity of rhein.

As the prevalence of CKD increases globally, the foremost effort of therapy is to prevent or delay the progression of renal diseases [26]. One principal pathological characteristic of obstructive nephropathy is renal interstitial fibrosis, regarded as a final step common for almost all types of CKD. Data generated from the current study indicates that p-STAT3 is likely to play an essential role in the progression of renal interstitial fibrosis in CKD resulted from various renal diseases. Our data also revealed that treatment with rhein suppressed interstitial fibrosis as well as apoptosis in a UUO rodent model. The findings present new evidence regarding the attenuation of CKD through angiotensin receptor. Rhein effectively reduces interstitial fibrosis as well as apoptosis in the UUO rat model employed in this study. The anti-fibrotic effect of rhein involves suppression of the STAT3 signaling pathway, which is associated with apoptosis of tubular cell and renal fibrosis. Additional investigations are necessary to evaluate the effects in humans, as well as to identify other potentially involved molecular pathways under rhein influence, but data from the present report are in agreement with the note that blockage of angiotensin receptor could be a first-line therapeutic option for patients suffering from chronic obstructive nephropathy.

## Conclusion

To conclude, the current study demonstrates that rhein exerts negative regulations on renal interstitial fibrosis to reduce UUO-induced renal fibrosis. Its curative effect is, at least partly, mediated via suppressing the apoptosis of tubular cells. In this regard, the current study not only highlights the note that inhibiting tubular cell apoptosis is likely a novel therapeutic option for treating fibrotic diseases, but at the same time provides a basis for using rhein against renal fibrosis.

## Data Availability

All data generated or analyzed during this study are included in this published article.

## Abbreviations

UUO: unilateral ureteral obstruction
α-SMA: α-smooth muscle actin
CKD: chronic kidney disease
